# Developing open source, self-contained disease surveillance software applications for use in resource-limited settings

**DOI:** 10.1186/1472-6947-12-99

**Published:** 2012-09-06

**Authors:** Timothy C Campbell, Charles J Hodanics, Steven M Babin, Adjoa M Poku, Richard A Wojcik, Joseph F Skora, Jacqueline S Coberly, Zarna S Mistry, Sheri H Lewis

**Affiliations:** 1 Johns Hopkins University Applied Physics Laboratory, 11100 Johns Hopkins Road, Laurel, MD 20723, USA; 2 Sotera Defense Solutions, Inc., 7230 Lee Forest Drive, Columbia, MD 21046, USA

**Keywords:** Electronic biosurveillance, Software development, Public health, Disease outbreak, Resource-limited settings

## Abstract

**Background:**

Emerging public health threats often originate in resource-limited countries. In recognition of this fact, the World Health Organization issued revised International Health Regulations in 2005, which call for significantly increased reporting and response capabilities for all signatory nations. Electronic biosurveillance systems can improve the timeliness of public health data collection, aid in the early detection of and response to disease outbreaks, and enhance situational awareness.

**Methods:**

As components of its *Suite for Automated Global bioSurveillance* (SAGES) program, The Johns Hopkins University Applied Physics Laboratory developed two open-source, electronic biosurveillance systems for use in resource-limited settings. OpenESSENCE provides web-based data entry, analysis, and reporting. ESSENCE Desktop Edition provides similar capabilities for settings without internet access. Both systems may be configured to collect data using locally available cell phone technologies.

**Results:**

ESSENCE Desktop Edition has been deployed for two years in the Republic of the Philippines. Local health clinics have rapidly adopted the new technology to provide daily reporting, thus eliminating the two-to-three week data lag of the previous paper-based system.

**Conclusions:**

OpenESSENCE and ESSENCE Desktop Edition are two open-source software products with the capability of significantly improving disease surveillance in a wide range of resource-limited settings. These products, and other emerging surveillance technologies, can assist resource-limited countries compliance with the revised International Health Regulations.

## Background

Emerging public health threats often originate in countries that lack many public health resources and infrastructure [1]. Because of the rapidity with which these diseases can spread, particularly with international air travel, early detection of disease outbreaks is extraordinarily important because it can provide for a quicker response and potentially limit morbidity, mortality, and the spread of the outbreak. In recognition of this fact, the World Health Organization issued revised International Health Regulations in 2005 (IHR 2005) that took effect in 2007. The purpose of IHR 2005 is to enhance global cooperation and protect populations from emerging health threats [2] by requiring participating countries to “develop core capacities for surveillance, detection, reporting and response.” These core capacities include legislation and financing, national and international communication, preparedness, human resources, and laboratory resources. The 195 member nations of the World Health Organization had until the summer of 2012 to comply with IHR 2005, which requires establishing capabilities for detecting, reporting, and assessing public health events involving a disease that would be a public health emergency of international concern. [3]. These requirements may be challenging for resource-limited countries, but there are ways in which technology may help. In 2005, Fraser et al. [4] described a practical guide for implementing electronic medical record systems using open standards and open source software, based on pilot projects in six developing countries. Recently, Were et al. [5] described a scalable open source electronic health record (EHR) implementation model that relies upon a national technical expertise center for external support and maintains electronic health records at multiple sites in resource-limited settings. Dennehy et al. [6] described a partnership model for electronic health records in resource limited primary care settings. Ashar et al. [7] described a variety of information and communications technologies that can be used for electronic health data capture and assessed their use in resource-limited settings. In 2010, Hartley et al. [8] described the Global Health Security Initiative and discussed how electronic biosurveillance systems complement traditional public health surveillance to provide early warning and international awareness of disease outbreaks.

Syndromic surveillance systems typically use electronic, non-traditional, pre-diagnostic health indicators as surrogates for disease incidence to detect potential outbreaks in populations [9]. These indicators may include a wide variety of data sources [10], such as over-the-counter and prescription drug sales data, emergency department visit chief complaint data, physician office visit insurance claims data, nurse hotline data, etc. Syndromic surveillance systems complement traditional public health surveillance by providing non-specific yet early pre-diagnostic indications of potential disease outbreaks [11]. The Electronic Surveillance System for the Early Notification of Community-based Epidemics (ESSENCE) is one example of an automated syndromic surveillance system. ESSENCE is a Java-based application used to monitor the health of populations and to detect disease outbreaks early and help prevent their spread [12]. The fully-functional web-enabled version of ESSENCE (called Enterprise ESSENCE) is used by local and regional public health departments in different areas of the US and by the US Department of Defense and Veteran’s Administration [13]. Enterprise ESSENCE is capable of collecting and analyzing a variety of data types and sources, and uses multiple anomaly detection algorithms to flag unusually high counts of disease indicators or alerts that are difficult for a human observer to see due to the volume and rate of change. System users can view, parse, plot, and map results, and share selected information with other users. Biosurveillance systems for resource-rich environments, such as Enterprise ESSENCE, are designed to use automated electronic data feeds and are best suited to areas with stable internet access.

Many global disease threats, like the 2003 Sudden Acute Respiratory Syndrome outbreak, first appear in resource-limited areas where electronic health data feeds and internet access are relatively unavailable or unreliable. Therefore, two additional versions of ESSENCE have been developed [14]: ESSENCE Desktop Edition (EDE) and OpenESSENCE (OE). Unlike Enterprise ESSENCE which uses proprietary commercial products, EDE and OE utilize freely available open source software that provides several advantages in managing health data and performing medical surveillance in a variety of global communities. These advantages include wide scrutiny for quality assurance, low cost for acquisition and maintenance, and extensive user input on requirements, usage, and adaptability [15]. Examples of the utility of open source software in health records and biosurveillance include: the public health data and information exchange methodology developed by the US Centers for Disease Control and Prevention (CDC) [16]; an open source electronic medical record for implementation in developing countries described by Mamlin et al. [17]; implementation of the Shibboleth information exchange for biosurveillance described by Lambert and Leonhardt [18]; and an open source cyber-environment especially for disease surveillance described by Edwards et al. [19].

Working closely with local public health departments, as well as the US military in their role of collaborating with host country military partners participating in support of IHR 2005 [20] in resource-limited countries in Asia, Africa, and elsewhere, the Johns Hopkins University Applied Physics Laboratory (JHU/APL) obtained potential user input to determine a set of requirements for OE and EDE. These health departments desire an open source software system that would place a minimum burden on data providers, easily allow the user to tailor the system to their needs, and provide sustainability by allowing local jurisdiction to control their data, minimize costs, and easily maintain the system. Table 1 compares the features of Enterprise ESSENCE, EDE, and OE based upon these user requirements.

**Table 1:** Comparison of features of Enterprise ESSENCE, OpenESSENCE (OE), and ESSENCE Desktop Edition (EDE)

**Feature**	**Enterprise ESSENCE**	**EDE**	**OE**
Uses proprietary 3^rd^ party software	X		
Deployable using only open source software		X	X
Supports multiple types of data	X	X	X
Supports a variety of data sources	X	X	X
Plug-in API* for detection algorithms	X	X	X
Security and encryption built into the core system	X		X
Customizable, dynamic, flexible design (property file driven)	X	X	X
Designed for internet access	X		X
Supports language internationalization			X
Supports font internationalization			X
Configurable security/access authentication	X		X

*API - Application Programming Interface.

EDE is a desktop version of ESSENCE that was developed for resource-limited and disaster settings with little or no internet access. EDE runs on a stand-alone computer as a self-contained application designed to deploy easily and function in diverse settings. EDE supports open development and extensibility. Although it does not have a built-in data collection capability, EDE easily reads a variety of data file formats, and allows the user to rename and characterize input variables. In the Philippines and Asia, EDE data have been collected by personnel entering data directly into electronic clinic records and via simple short message service (SMS) text messaging. Other countries plan to use smart phone data forms to collect health data and transmit it via SMS.

As the name implies, OpenESSENCE (OE) is an open source application that includes key features of Enterprise ESSENCE and can be used either with the internet or as a stand-alone system [14,21]. OE does not require the automatic secure internet data feeds that are used in Enterprise ESSENCE. Because of the distinct differences in Enterprise ESSENCE and its user community, converting all instances of Enterprise ESSENCE to OE is not planned at this time. Like EDE, OE provides for open development and extensibility, however, unlike EDE, it also contains a built-in data entry module. Data can be entered directly into the OE server, or via the web by multiple, geographically distributed users.

It should be emphasized that the primary goal of these efforts is to build capacity by giving resource-limited countries the independent ability to collect and analyze their own data. Because of this, JHU/APL does not have access to their data nor is that a goal. Based on more than a decade of experience working with local public health officials within the US, JHU/APL interacts closely with local public health officials in these countries in order to address quickly their concerns about best utilizing and maintaining their new system. The emphasis is on rapidly providing value as recognized by the user so that they begin using these systems as much as possible.

## Methods

OE and EDE were developed to support a wide variety of user needs ‘out of the box,’ including configuration for different data sources and for user-performed queries on that data. EDE can be downloaded from the web and loaded onto most Windows-based laptop or desktop computers. Development is not yet complete on OE, so for the moment it requires active installation by JHU/APL developers. Eventually, we intend to offer the OE version as a downloadable product. The biosurveillance system results are provided in a format similar to Enterprise ESSENCE [12,13] and include graphs, charts, and detailed data on case reports, as well as geographic maps of individual illness reports.

### OpenESSENCE (OE)

#### Overview of software framework

Security, encryption, and user-interface language internationalization support are built into the core OE system. OE uses open source software and industry standard technologies (e.g., Java EE, Apache Tomcat, Spring, MySQL, PostgreSQL, PostGIS, GeoServer, GeoExt, Openlayers). The overall design is modular with component-based applications and a data-driven system structure. OE utilizes a Java development open source framework called Spring [22,23]. Spring contains modular features for developing web applications in Java. These features include aspect-oriented programming, transaction application programming interface (API), configurable security processes for authentication and authorization, the use of XML schema, and data access for working with relational databases. OE also uses Groovy [24,25], an object-oriented programming language that enhances and interoperates with the features of Java. Groovy is used to implement the interface layer for the data sources, allowing dynamic table and field definitions. Spring provides comprehensive support for using classes and objects defined by Groovy. This OE design allows for dynamic extension and reconfiguration, and eliminates the need to re-develop or rebuild an application in order to incorporate adjustments or enhancements. Plug-in APIs are used for the detection algorithms. Various libraries and server applications are stable.

#### Initial user interface and data input

**Figure 1:**
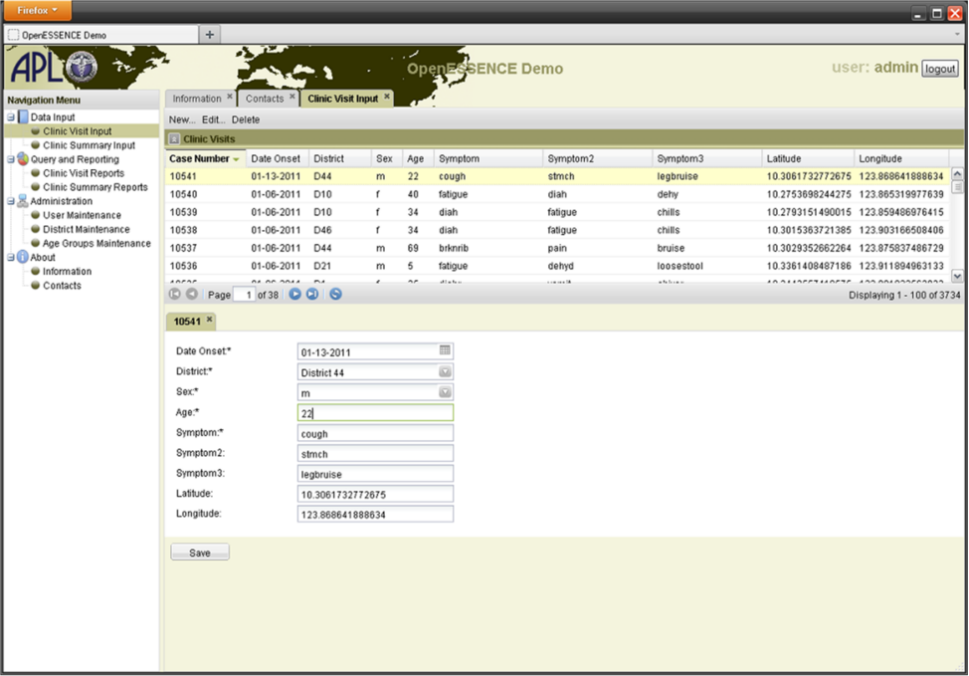
User interface for entering data into OE.

Health indicator data reflect determinants of health and health outcomes and may come in a wide variety of formats that include numeric and text data. OE is designed to accept a variety of types of data input [26]. One method of data input is through the web data entry user interface. Figure 1 illustrates the user interface for entering various types of data. Note that OE supports language internationalization of all user interface components. The data can be input by typing free text, including numeric data, as well as the different selections from the pull-down list. The free text data can be validated with configurable data parameters, such as maximum/minimum ranges, decimals, etc. The software is configurable to adjust for different databases and data sources, and can accept combinations of different data sources (e.g., joins of multiple tables). For data input through a database connection, standard extract, transform, and load (ETL) synchronization processes can be used to load surveillance data from any Java Database Connectivity (JDBC) compatible database.

In many resource-limited countries, cellular telephone usage is already or is rapidly becoming very common [27]. These services often include the ability to transmit text messages or SMS. Therefore, SMS messages can be used as a way of collecting health data and entering it into an electronic surveillance system [7,28]. JHU/APL is piloting such systems in Asia and Central America.

**Figure 2:**
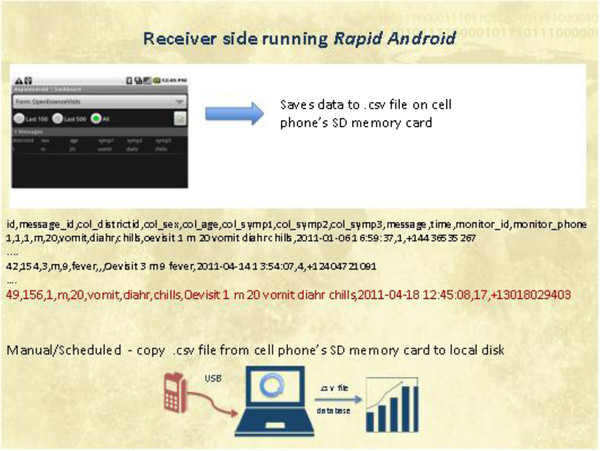
Example of record format and data flow for cell phone inputs into OE.

In addition, interactive voice response (IVR) has been used as a method of electronic collection of health data [7]. IVR can be used with both cellular and land telephone lines. Figure 2 shows an example of how health data may be electronically collected by OE using cellular telephones. In this example, the user enters data using IVR at the time of the encounter and these data are then stored securely on the device until they can be transmitted to the central system. JHU/APL has developed and is piloting these data entry systems in Peru.

#### Analysis and visualization

Tools to analyze and visualize data and derived data products are important ways in which biosurveillance systems can enhance public health decision making. It is important to note that OE provides the ability for multiple users to share information derived from data without having to share sensitive private health data. Such ability enhances the sharing of information across jurisdictional boundaries.

**Figure 3:**
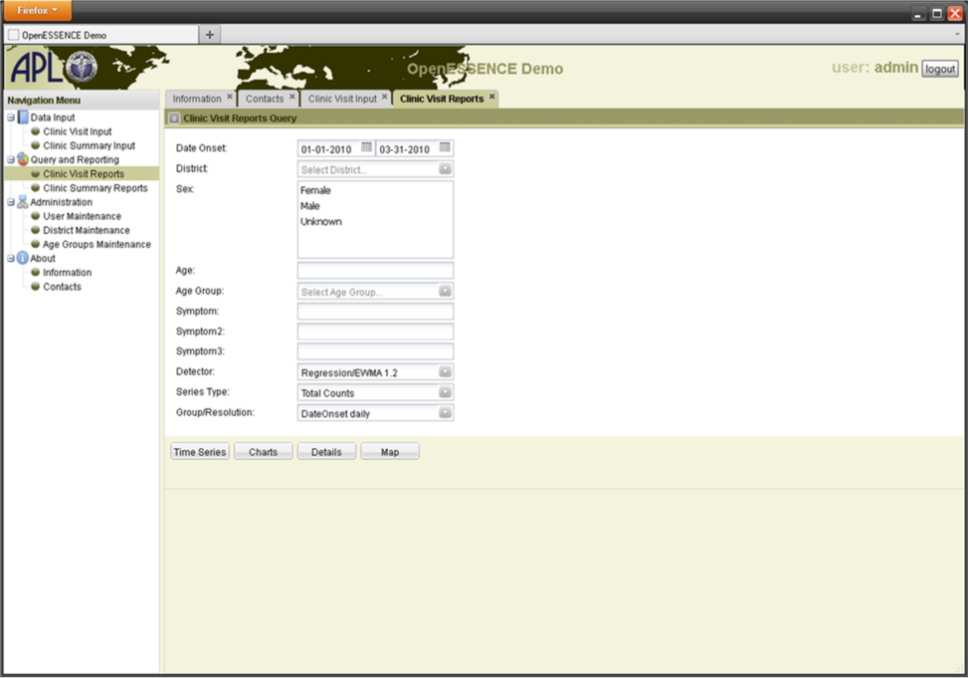
**Screen shot of the data report query form for OE.** Note that the user can select which detector algorithm they wish to use.

Queries are used to sort and filter the data for analysis. The query form, including a selection for detector algorithms, is shown in Figure 3. Stock filter fields include date range, selection from list, multi selection, free form text/number, etc. The free form text input fields support database wildcards. Combinations of logical “AND” and “OR” operators can be used in the query. As in Enterprise ESSENCE, queries can be built using a graphical user interface so that a detailed knowledge of structured query language (SQL) is not needed. This query builder approach focuses all database access logic into one part of the code base, thereby making it easier to manage. Because these queries may be saved and bookmarked, they can be used to establish case definitions so that multiple users may analyze their data in a similar fashion and share results for comparison.

**Figure 4:**
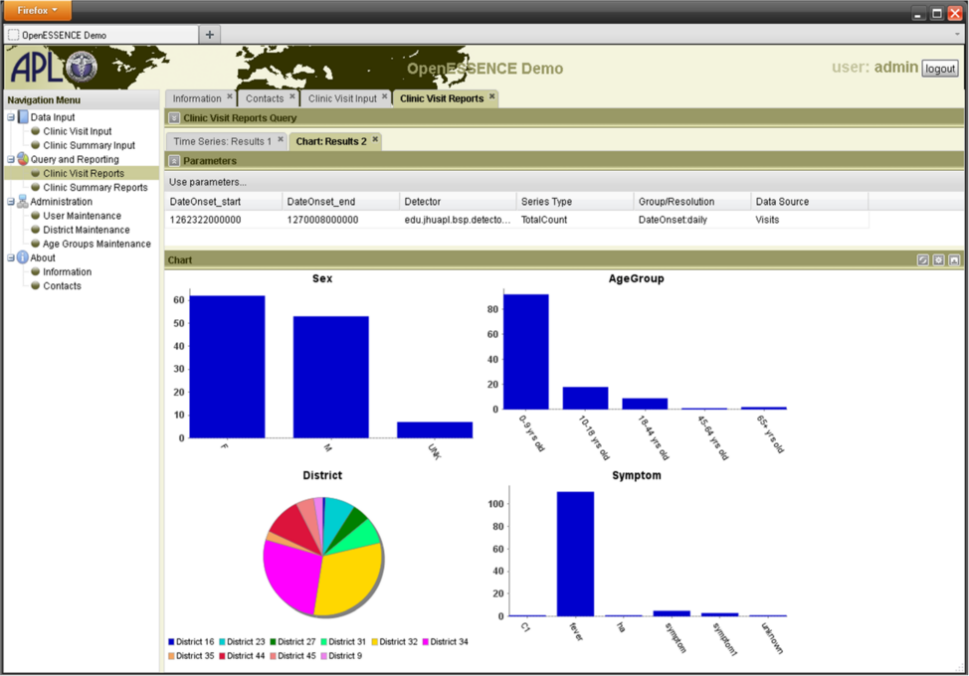
OE screen shot illustrating configurable charting, pie/bar over configured groupings.

Figure 4 is a screen shot showing how data can be analyzed by examining different categorizations of data relationship. In this example, the data groups are: Sex, Age Group, District and Symptom. Such features may allow the user to determine if the disease outbreak is more prevalent within or among different data groups. Data sources are more sensitive to outbreak detection if they carry information that can be used to group persons potentially affected by an event and to exclude those unaffected.

**Figure 5:**
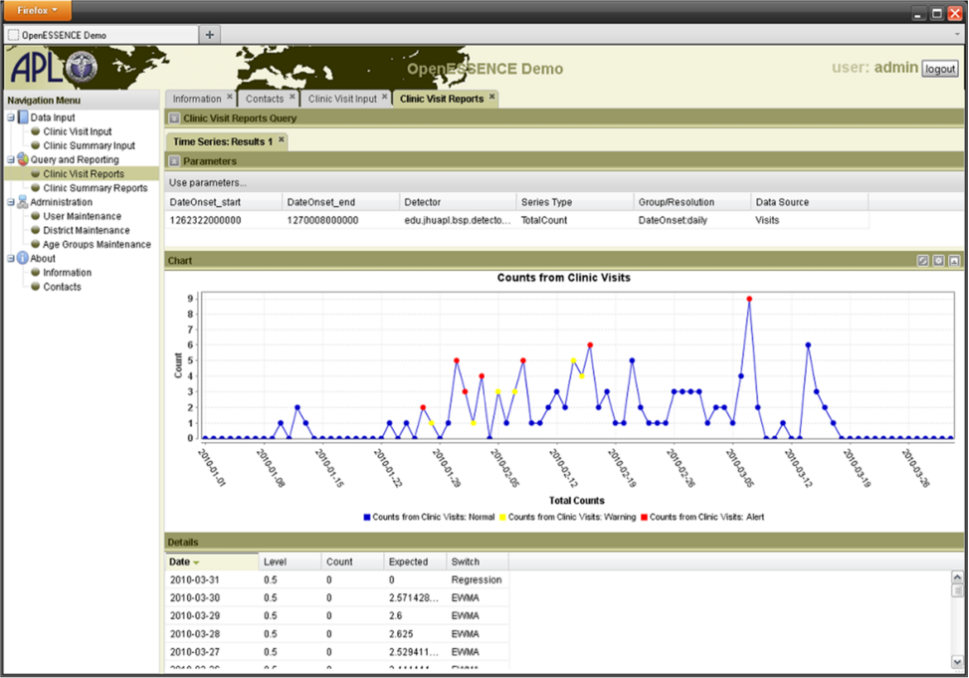
**OE screen shot of time series analysis.** Below the plot are details of the detection algorithm output. Note that detection algorithm alerts can be shown by the red peaks in the plot.

Figure 5 is a screen shot of a time series showing detection analysis. Clicking on the points on the time series plot shows the user a detailed view of the records. OE uses a plug-in API for detection algorithms and includes a selection of “none” to disable use of a detection algorithm. Currently provided algorithms include Exponentially-weighted Moving Average (EWMA), linear regression, Poisson regression, and the US Centers for Disease Control and Prevention (CDC) Early Aberration Reporting System (EARS versions 1, 2, and 3). Additional detection algorithms can be implemented using the plug-in API. Time series visualization includes anomaly detection and image customization and export. These features assist users in sharing information while investigating a possible outbreak.

**Figure 6:**
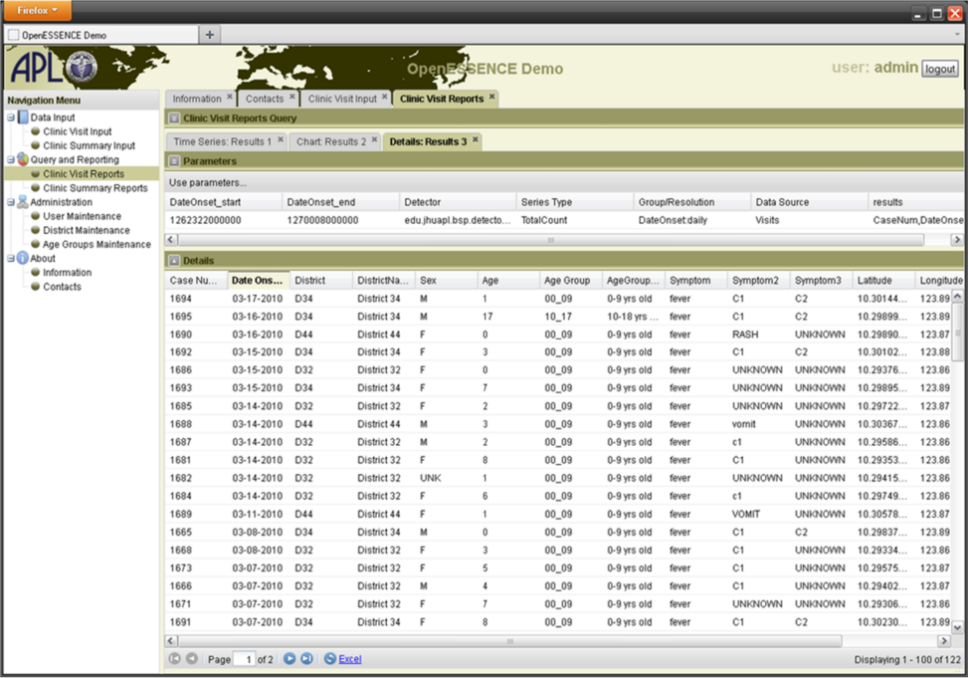
Screen shot of an example of data details analysis available in OE.

Figure 6 is a screen shot of a data details analysis. By allowing the user to “drill down” into the data, users can examine the specific health information that is resulting in an algorithm-derived alert. Data details, including column ordering and sorting, can be exported to Microsoft Excel and/or comma-separated variable (CSV) files for further analysis using other tools.

**Figure 7:**
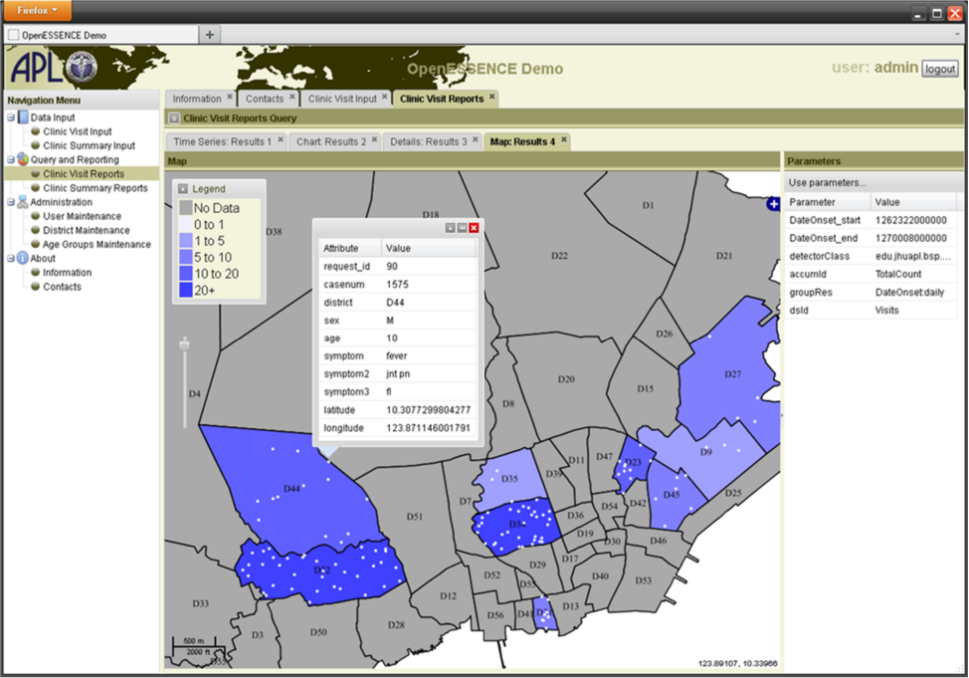
OE screen shot of geographic information system (GIS) map of data results.

Figure 7 is a screen shot showing analysis of data by geographic region. The output of different data queries and detection algorithms may be used to create maps of specific types of information. For example, there are detection algorithms that can be used specifically for geographic data, such as SatScan that analyzes space-time data [29-31]. Therefore, a user can determine whether a disease is occurring in clusters or is randomly distributed. This allows the user to determine quickly which geographic regions are primarily impacted and to look for geographic outbreak patterns. The ability of OE to export such geographic images may assist users in sharing information while investigating a possible outbreak.

### ESSENCE desktop edition (EDE)

#### Overview of software framework

EDE utilizes the Eclipse Rich Client Platform (RCP), an industry standard open tools platform used for a variety of information technology applications [e.g., [32]. This is a customizable platform built with software units called ‘plug-ins’ that support modular development. Plugins are designed to add specific capabilities to an existing software application in a convenient and user-friendly way. The plug-ins register themselves with and utilize the services of the existing application and allow developers to add functionality, upgrade features, and deploy bug fixes. EDE consists of three primary plug-ins:

1) desktop core, which provides the main user interfaces;

2) desktop data core, which provides the underlying data query mechanism;

3) detector temporal core, which provides detection algorithm interfaces.

#### User interface and data input

**Figure 8:**
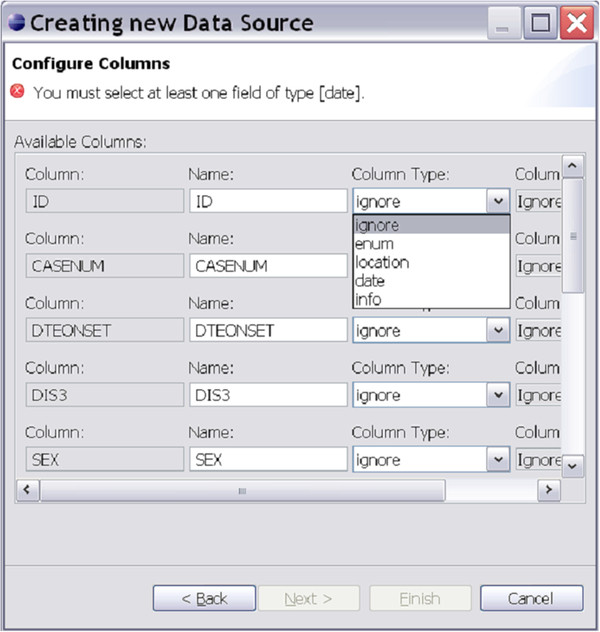
Configuration wizard for adapting EDE data input to type of data.

The EDE configuration wizard allows the user to configure EDE to support their data, rather than vice versa, thereby minimizing difficulties encountered when manipulating a dataset to enable ingestion by an analysis program. Figure 8 shows the data source creation wizard where users configure EDE to match their dataset. The data source configuration in EDE supports multiple database systems, including Microsoft Access, Apache Derby, Microsoft SQL Server, Microsoft Excel, PostgreSQL and delimited text files.

#### Analysis and visualization

**Figure 9:**
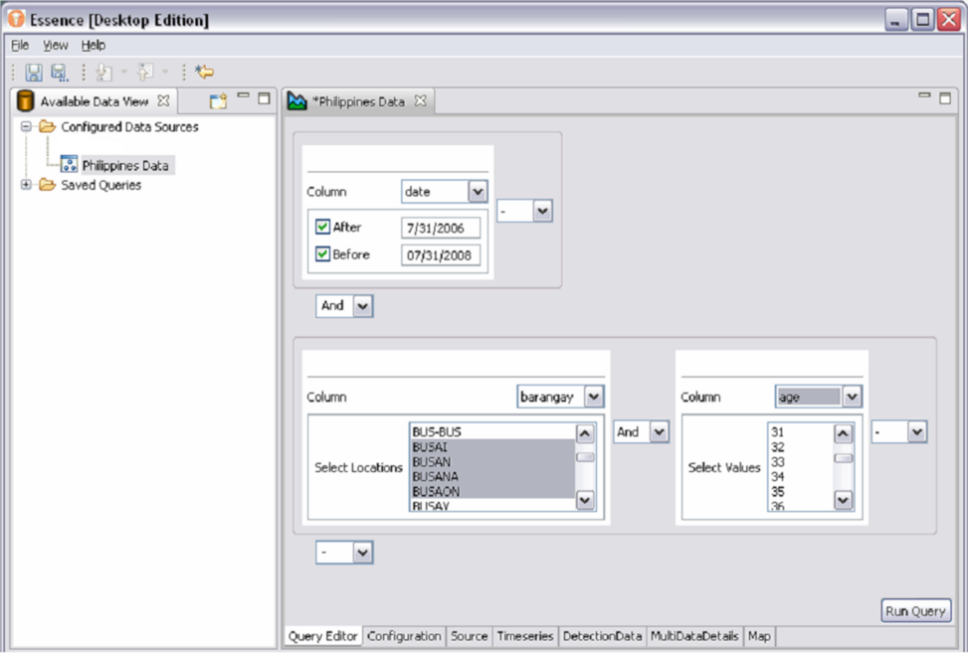
Example of the user interface for creating a query in EDE.

As with OE, the analysis and visualization tools in EDE are data agnostic, meaning that they can be syndromic health data, diagnostic data, sensor input, EHR, or even non-medical data. EDE data queries can be created by the user, performed and saved for future use. These queries are built graphically using a dynamic interface. This interface allows the user to perform grouping queries using “AND” and “OR” conditions allowing complex logic such as “(a AND b) OR (c AND d),” which is difficult or impossible with some graphical query tools. Figure 9 illustrates the user interface for query creation.

**Figure 10:**
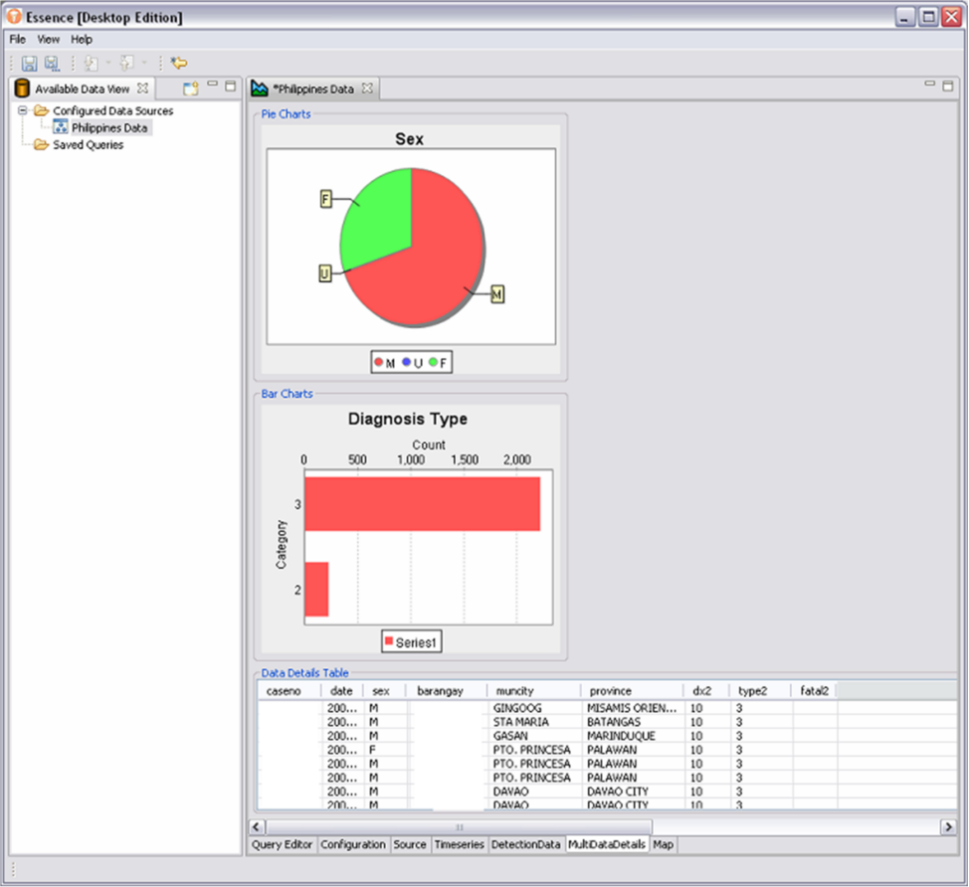
EDE allows the user to examine the data in different slices, such as by sex or by diagnostic code type.

When executed, the query created in Figure 9 produces a time series plot, data details, graphics, and maps that can be used to examine the population subset described by the query. Figure 10 shows an example of how different slices of the data can be visualized. In addition, different types of detection algorithms can be selected for a query. Currently provided algorithms include Exponentially-weighted Moving Average (EWMA), Gstat (an open source computer code for multivariable geostatistical modelling, prediction and simulation), linear regression, Poisson regression, and the US Centers for Disease Control and Prevention (CDC) Early Aberration Reporting System (EARS versions 1, 2, and 3). In addition, EDE allows developers to add other algorithms through the use of RCP plug-ins. Using the Eclipse RCP plug-in framework, these added algorithms integrate seamlessly into the EDE application.

**Figure 11:**
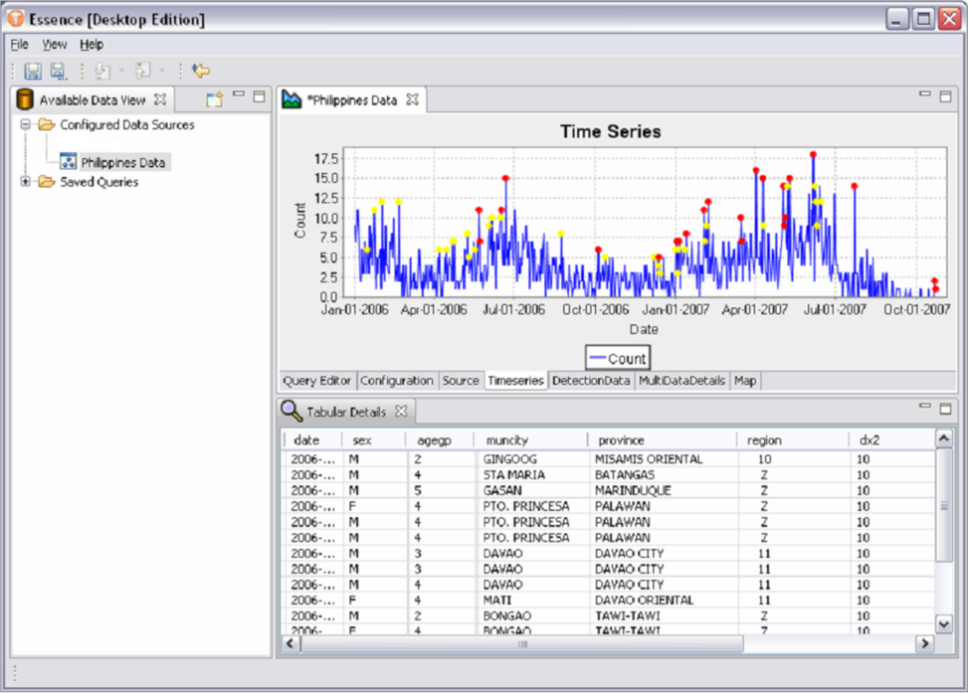
An example of a time series plot generated by EDE Note the red and yellow dots indicating different types of user-defined alerts.

Figure 11 shows an example of a time series plot generated by EDE. The red and yellow markers correspond to red and yellow alerts as determined by the detection algorithm selected by the user. The user can hover over the marker to get tooltip information regarding the detection alert. The user can also click on a marker to highlight that data point, which then updates data detail views allowing the user to investigate further the data point.

**Figure 12:**
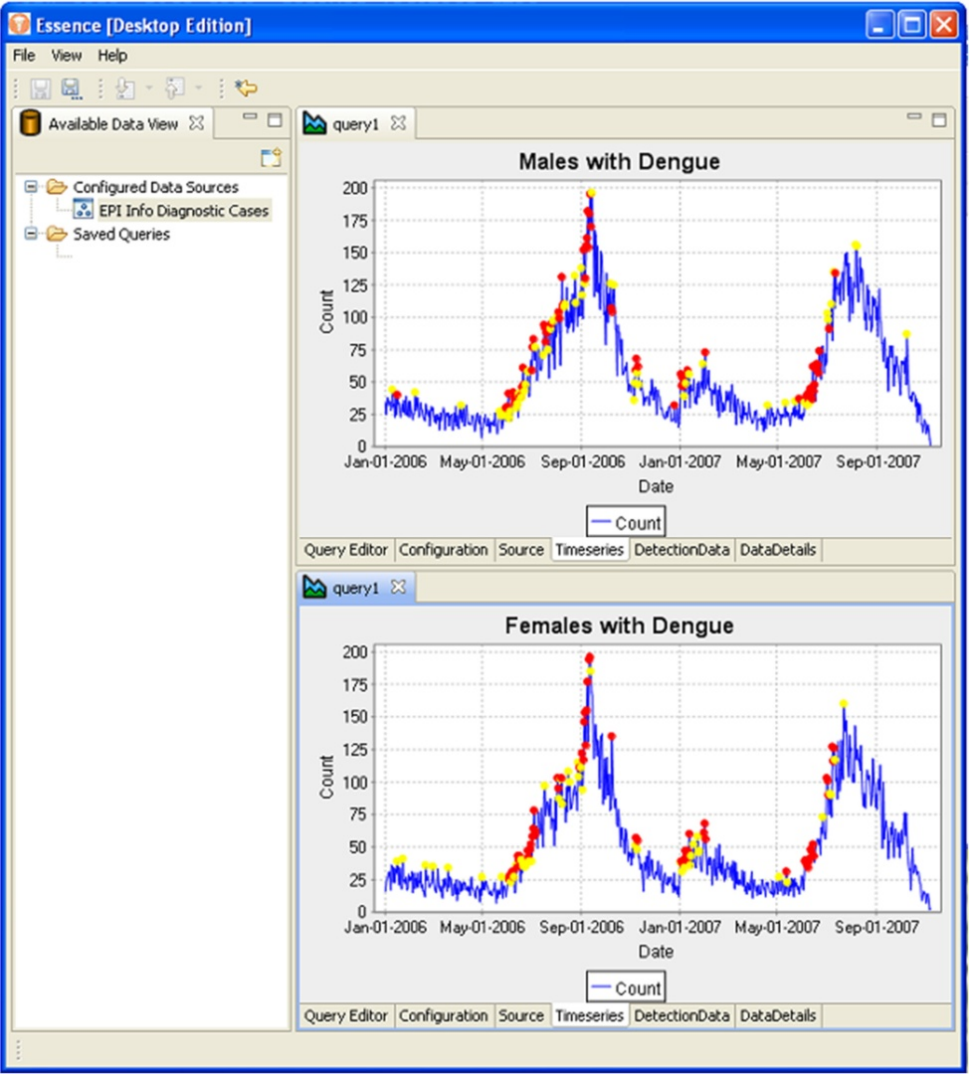
EDE screen shot illustrating the results of multiple data queries and how comparisons may be made.

EDE allows the user to configure the look and feel of the application to suit their perspective and analytical style. This includes having multiple queries displayed concurrently. Figure 12 illustrates how the user can drag and arrange windows as they choose.

**Figure 13:**
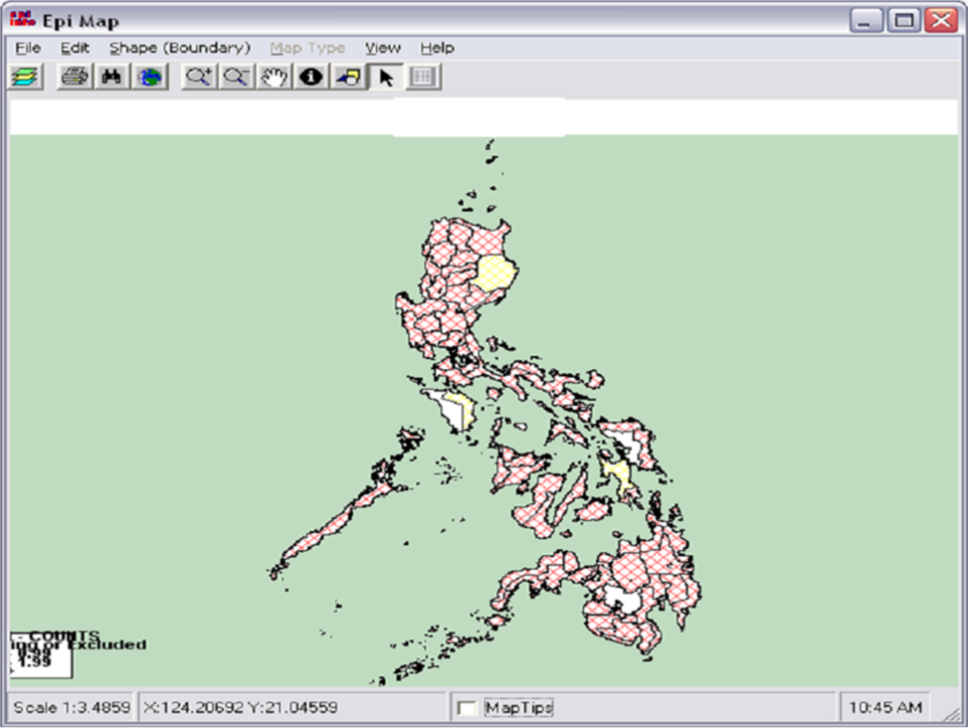
EDE can be used with Epi Info’s Epi Map for geographical display of data.

EDE also provides a plug-in framework for mapping geospatial data. This framework includes mapping with Epi Info Epi Map [33] as shown in Figure 13. The US CDC developed Epi Info for use by physicians, nurses, and epidemiologists to collect public health data for statistical analyses. Epi Info includes the Epi Map module to display geographic maps of data utilizing the Environmental Systems Research Institute (ESRI) MapObjects software. EDE will launch Epi Map for a specific query and allow the user to customize the map.

In addition, EDE supports the Desktop Internet Geographic Information System (uDig) mapping application, which is an open source Java-based geospatial data viewer/editor toolkit [34] built with Eclipse RCP. As with EpiMap, uDig can be launched for a specific query and the map appearance customized by the user. uDig also allows developers to customize and integrate geographic information systems (GIS) applications, including ESRI shape files, PostGIS, and geospatial web services.

## Results

The OE system was only recently deployed in 2011 and results are not yet available, but the EDE system has been in use long enough for preliminary results to be presented. The initial deployment of EDE was as an add-in module attached to the national disease surveillance system in the Philippines called the Philippines Integrated Disease Surveillance and Response (PIDSR) program. The goal of PIDSR is to reduce morbidity and mortality through a nationwide system that integrates facility-based information systems. EDE is now integrated with PIDSR and is used to monitor the temporal trends of diseases that are officially notifiable in the Philippines. Epidemiologists and computer scientists from JHU/APL visited the Philippines to solicit input from the stakeholders about the system architecture, types of usage, means of inputting data, and training. In 2009, a pilot study was begun to evaluate a simple fever surveillance protocol using SMS text messages to send daily, patient-level data from several local health clinics to the city health office in Cebu City [28]. These data were included in a single SMS text message for each patient who presented with fever at the local clinics. Family and address codes, age, sex, date of onset, and presenting signs and symptoms were recorded for each patient as per the usual protocol. A formatted SMS text message about each eligible patient was sent each day to a receiver phone connected to a computer at the city health office. Standardized abbreviations were adopted for specific signs and symptoms (e.g., ha = headache). An SQL application was used to download the SMS data from the phone to an Epi Info database, which was analyzed using EDE by the city epidemiologist. Recently, the local health department has expanded SMS data inputs to all of Cebu City.

Before implementation of the SMS system, there had been a minimum two weeks delay between case presentation and case reporting to the city health office. SMS text messaging was the only practical alternative for this particular location because none of the local health clinics had an operable computer or internet connection. SMS was inexpensive and commonly used in this region. Using SMS also allowed the currently used logbook format to be maintained. Within a month of implementation of fever case data collection, 30 % of the local health clinics were using SMS texting to send daily fever case reports to the city health office. Within two months of implementation, this usage expanded to 90 % of the local health clinics and so far remains at about that level. The primary obstacle to increasing usage has been that local health department personnel have been accustomed to monitoring data post-event or at weekly or longer intervals based on previous data availability. Now that they have the ability to do daily monitoring, it can take some time for the users to become accustomed to doing so.

## Discussion and conclusions

Based on information obtained from biosurveillance stakeholders in resource-limited countries, JHU/APL learned the following lessons: 1) the ability to maintain control of one’s own data is very important to the users, 2) open-source software is particularly desirable, 3) the system should place a minimum burden on those providing data, 4) system acceptance results from enabling the user to easily tailor the system to local needs, and 5) sustainability results from local ownership and working within existing needs and capabilities. This list of points is based upon informal qualitative discussions with local users regarding their needs, as our primary focus has been on rapid technology insertion, refinement, and increasing usage capacity. In addition to these lessons, our experience has revealed the importance of interaction with the appropriate levels of the local and national governments and the identification of key individuals who can serve as champions of the project, including those involved in local policy and financial matters as well as the actual users.

Therefore, the OE and EDE systems were created by JHU/APL to offer self-contained disease surveillance tools that can be deployed efficiently at a variety of resource-limited locations, as well as disaster locations. Both these systems are easily upgradeable and extendable. While skilled information technology professionals may be difficult for public health departments to find and retain, the OE and EDE systems have been simple enough to operate that this has not been a significant limitation. EDE was designed as a stand-alone desktop application, and OE can be used as a desktop or web-based application. Each provides similar functionality to the current web deployment of Enterprise ESSENCE [12]. Both systems are based on a modular, component-based application design. This design allows for improved testability of components and isolation of problems. Repurposing and reuse is also easier and more likely with a modular design because it mitigates the need to redevelop or rebuild an application to incorporate adjustments or enhancements. The system structure is data driven to allow for dynamic extension and reconfiguration.

These biosurveillance systems were developed to support a wide variety of user needs in different settings. Both systems will provide user-defined preferences and mechanisms for data input from several types of databases. Users can configure the system specifically for the variables included in their database, thereby easing common data ingestion problems, especially the difficulties in trying to get disparate data formats to fit a specific type of data ingestion. Users can design their own queries and use different detection algorithms to analyze their data, including temporal and spatial analysis.

EDE utilizes an Eclipse RCP framework, while OE uses the Spring framework and Groovy Java language extensions. While both systems can operate on a single computer without a network connection, the OE system is designed to utilize the benefits of network connectivity. The OE system can also be used to share actionable information via the internet among multiple users and across different jurisdictions. Both systems provide results in a format similar to Enterprise ESSENCE [12], including graphs, charts, detailed data on individual illness reports, and geographic maps of locations of individual illness reports. As open source stand-alone desktop applications, they are easily deployable, upgradeable and extendable. These features make EDE and OE ideal for rapid deployment in resource-limited environments where the infrastructure for a full-scale web-based biosurveillance system is not immediately feasible. Future efforts are to continue to improve the software features available to the user, while keeping the interfaces and components as simple as possible so that maintenance and sustainability do not require someone with a high level of information technology expertise. Such biosurveillance systems may then improve the timeliness of data collection and enhance the early detection of disease outbreaks, thereby allowing time for mitigation of the effects of these outbreaks.

## Competing interests

The authors declare that they have no competing interests.

## Authors’ contributions

TCC was the technical lead and developer for the OE application, and helped develop EDE. CJH was the technical lead and developer for the EDE application and helped develop later versions of OE. SMB wrote the manuscript, selected figures, and obtained references, based on his work on the ESSENCE project. AMP was the technical lead for telephone and SMS data ingestion design and development and was a software developer for OE. RAW was the chief software engineer for all versions of ESSENCE. JFS was the original software technical lead and software developer for OE. JSC was the chief epidemiologist on the project and obtained user input and usage data on the application. ZM developed several key components on the EDE project. CJH, TCC, RAW, JSF, JSC, and SHL helped develop user requirements for these applications. All authors read and approved the final manuscript.

## Disclaimer

The views expressed here are the opinions of the authors and are not to be construed as official or as representing the views of the US Department of the Navy or the US Department of Defense.

## Pre-publication history

The pre-publication history for this paper can be accessed here:

http://www.biomedcentral.com/1472-6947/12/99/prepub
